# Registered professional nurses’ experiences regarding supervision of nursing care at selected hospitals of Limpopo province

**DOI:** 10.4102/curationis.v49i1.2846

**Published:** 2026-04-15

**Authors:** Munyadziwa R. Raliphaswa, Takalani R. Luhalima, Julia L. Mafumo

**Affiliations:** 1 Department of Advanced Nursing Science, Faculty of Health Sciences, University of Venda, Thohoyandou, South Africa

**Keywords:** patient safety, patient outcomes, registered professional nurses, quality nursing care, supervision and nursing care

## Abstract

**Background:**

Registered professional nurses are the most qualified category in the nursing profession and are mandated by the scope of practice to supervise other nursing categories during the provision of nursing care. Nursing supervision is a cornerstone of high-quality nursing care that must be integrated into daily nursing tasks to ensure patients receive optimal treatment and minimise patient complaints and lawsuits.

**Objectives:**

The objective of the study was to explore the experiences of registered professional nurses regarding supervision of nursing care in the selected hospitals of Limpopo province.

**Method:**

A qualitative study was conducted using an exploratory, descriptive and appreciative inquiry design. The principles of appreciative inquiry served as a guide for the data collection method. Twenty-four registered professional nurses from the maternity, paediatric and casualty departments of selected hospitals were interviewed. Data were gathered through semi-structured individual interviews using standardised interview protocols. Sample size was determined by data saturation. Though data saturation was reached at participant number 15, the researcher kept going on. In order to authenticate the findings, three participants were added from each selected hospital and clinical area.

**Results:**

The emerging themes included the administrative, organisational, educational and training challenges.

**Conclusion:**

Nursing care supervision by registered professional nurses is essential to improve the quality of nursing care and levels of satisfaction.

**Contribution:**

This study contributes to the understanding of factors that impact of effective supervision of nursing care by professional nurses and outline the areas of improvement. The improvement in supervision of nursing care may lead to reduction of patients complaints, improved patient safety and uplift the standard of nursing care rendered.

## Introduction

Supervision of nursing care encompasses both clinical and administrative oversight of nursing care, as well as supervision of all nursing procedures and general nursing practice (Raliphaswa et al. 2021). The person responsible for supervising nursing care in the clinical area is the registered professional nurse, as mandated by the nursing regulations *Act no 33 of 2005* (Republic of South Africa 2005). The Scope of Practice of registered professional nurses (R2127) described the duties of registered professional nurses in clinical practice, which include managing nursing care through nursing care supervision and providing comprehensive nursing care (SANC 2022). Nursing care supervision is compromised by the demanding and challenging work environment in clinical areas. Inadequate nursing care oversight puts patient safety and nursing care quality at risk, sometimes leading to care being overlooked or compromised.

Nursing care is a vital component of healthcare, and the quality of a hospital’s nursing care can significantly impact its reputation. The cornerstone of nursing services is the care that nurses provide to patients, their families and the broader nursing community (Alwani 2020). The registered professional nurses supervise nursing tasks, which include patient bathing, feeding, assisting with elimination needs, maintaining a comfortable and clean environment, wound care, administering medications to patients and ensuring adherence to protocols.

According to Snowdon et al. (2020), the Proctor Supervision Model is designed to help and guide registered professional nurses in supervising nursing care, thereby improving patient and clinician outcomes. Kustiati, Pohan and Hartiti (2021) further state that providing nursing services to individuals, families, groups or communities is what nursing is all about. High-quality nursing management will yield high-quality nurses, which in turn will deliver high-quality nursing services.

Nonetheless, it appears that many registered professional nurses were not sufficiently prepared to supervise nursing care at the start of their employment, so their capacity to supervise nursing care may vary (Mabusela & Ramukumba 2021). International supervision of nursing care was carried out with passion and diligence; however, in the African continent, supervision of nursing care is inconsistently applied due to a lack of training, shortages of nursing staff and limited material resources (Atashi et al. 2023). Locally, the supervision of nursing care is carried out differently because the challenges are not the same, depending on the area whether rural or urban (Alhassan et al. 2025). Therefore, specific information and competencies must be taught in the traditional nursing school curriculum to ensure proper supervision of nursing care. Many patients’ complaints and legal actions have been influenced by instances of substandard nursing care resulting from inadequate nursing care supervision and negligence (Gcawu 2020). The complaints from these patients, along with the numerous lawsuits, prompted the researcher to investigate the experiences of registered professional nurses regarding the supervision of nursing care.

In the current literature, there are limited studies on the experiences of registered professional nurses regarding the supervision of nursing care locally; hence, the researcher’s interest in exploring the experiences of registered professional nurses regarding the supervision of nursing care at the selected hospitals in Limpopo Province.

### Objectives

To explore and describe the experiences of registered professional nurses regarding supervision of nursing care in the selected hospitals of Limpopo Province.

## Research methods and design

This qualitative study employed an exploratory, descriptive and appreciative inquiry design. A qualitative approach with exploratory and descriptive designs was employed to explore and describe the experiences of registered professional nurses regarding nursing care supervision. Appreciative inquiry is a generative process that has been applied worldwide at all organisations to bring cultural transformation and is an evidence-based approach to change and individual, social and organisational wellbeing (Armstrong, Holmes & Henning 2020). An appreciative inquiry design was used to ascertain what is and is not functioning well in an organisation with regard to nursing care supervision.

### Setting

This study was conducted in the province of Limpopo, which is made up of five (5) districts, namely, Vhembe, Capricorn, Waterberg, Mopani and Sekhukhune. Each district has one regional hospital that serves as a referral from the district hospitals, whereby very sick patients and those who need specialised care are referred. Three (3) districts, which are Vhembe, Waterberg and Mopane, were selected, and one regional hospital in each of the three (3) was selected. Limpopo Province has 22 health centres, 471 clinics and 43 hospitals to serve its sizeable population of 6 million people (Stats SA 2020). The province borders Zimbabwe to the North, Mozambique to the East and Botswana to the West. The province is predominantly rural, with many people facing unemployment and living below the poverty datum line (Molalakgotla 2020). This contributes to the increased burden of disease and high referral rate.

### Study population and sampling strategy

#### Study population

Brink, Van der Walt and Van Rensburg (2018) described a population as the whole set of people or things that the researcher is interested in, or that fit the requirements for the study. The study’s population comprised all categories of nurses, namely registered professional nurses, registered staff nurses and registered assistant nurses in Limpopo Province. The target study population included registered professional nurses from selected hospitals in Limpopo Province, because they were knowledgeable about the experiences of nursing care supervision.

#### Sampling strategy

According to Brink et al. (2018), a sample is referred to as a subset of the population that is selected to represent the population. Sampling techniques are methods used to select a subset of individuals or items from a larger population for analysis (Brink et al. 2018). The sample strategy employed in this study was non-probability, purposive sampling, as the selected participants possessed knowledge of the phenomenon and were chosen based on the researcher’s judgement (Brink et al. 2018).

Due to the nature of the workload and the patients who require treatment, the researcher purposefully chose the maternity unit, paediatric ward and casualty department. Additionally, the registered professional nurses were specifically chosen because they were well-versed in nursing care supervision and could thus provide the researcher with comprehensive information about supervision. Registered professional nurses with five or more years of experience are more experienced in supervising nursing care; therefore, they met the inclusion criteria (Mabusela & Ramukumba 2021). Potential participants were excluded if they did not meet the inclusion criteria. Even though the data were saturated at participant number 15, the researcher nevertheless continued with three participants from each hospital to confirm the results. As a result, the whole study included 24 registered professional nurses, aged 36–63 years old.

#### Data collection

Data collection describes the way in which the researcher approaches answering the research questions (Brink & Van Rensburg 2022:155). Because people are best understood in their environments, data were gathered in the settings where the participants worked, which were the maternity unit, paediatric ward and casualty department of the chosen hospitals. The nursing managers were responsible for granting permission to access the participants. The managers were not involved during the data collection process and never met with participants. Information was gathered from registered professional nurses who met the requirements at the chosen hospitals. The researcher met with the participants to explain the objective of the study and how the data would be collected. The researcher further explained that participation was voluntary, and participants provided written consent before the study was conducted.

Data were collected through semi-structured in-person interviews using interview guides from May to November 2024, as the researcher aimed to explore the experiences of registered professional nurses concerning nursing care supervision. This approach enabled the researcher to gather comprehensive and high-quality data about the experiences of registered professional nurses who supervise nursing care in selected hospitals in the province of Limpopo (Gray, Grove & Sutherland 2017). The interview guide was developed based on the Appreciative Inquiry design.

The operational managers of the chosen wards or units of the chosen hospitals planned the location. Informed consent was gained when the participants were informed about the study by the researcher. After obtaining the participants’ consent, a voice recorder was utilised to collect data. To document the participants’ informal behaviour, the researcher took field notes during the interviews. The semi-structured questions that were asked during the interview were:

- *What are your best aspects regarding the supervision of nursing care in your hospitals?*
- *What are the challenges that you are encountering when supervising nursing care in your ward and hospital?*
- *How best do you think you can inspire other registered professional nurses to improve the supervision of nursing care in your ward and hospital?*

The participants were invited to openly discuss their experiences with nursing supervision, and the researcher used probing to ensure that all topics were covered (Polit & Beck 2017). The participants were 24 registered professional nurses, and data saturation was reached at the 15th participant. The researcher continued with data collection by adding three (3) participants from each selected hospital and clinical area to authenticate the findings.

#### Data analysis

Data were collected through semi-structured face-to-face interviews using a voice recorder and field notes.

The researcher meticulously inspected and analysed the collected data and field notes, using Tesch’s eight steps, and the data were transcribed verbatim (Creswell 2014). The data analysis process, following Tesch’s steps, included several key actions: (1) Transcription and categorisation of interview data; (2) Repeated reading and commentary for insights; (3) Listing, clustering and arranging topics into main and subcategories; (4) Abbreviating topics into codes aligned with relevant text; (5) Labelling categories with descriptive phrases; (6) Standardising and organising abbreviations alphabetically; (7) Collecting and documenting data for each category; and (8) Final interpretation by group (Cresswell 2014).

The researcher examined the data multiple times to collect similar information regarding the experiences of registered professional nurses. The data were classified in a coherent and orderly manner, with similar themes grouped together. Data from each category in a location were grouped together and coded as themes. The supervisor assisted with the coding of the results. The researcher provided a summary of the themes and sub-themes. The raw data were also forwarded to an experienced independent coder for coding, and an agreement was obtained on the detected themes and sub-themes.

### Measures of trustworthiness

Lincoln and Guba’s model of trustworthiness, as discussed in Polit and Beck (2017), encompasses credibility, transferability, confirmability and dependability to ensure trustworthiness. To ensure reliability, the study ensured that procedures were followed and upheld. The researcher established credibility by engaging in extended interactions with participants through semi-structured in-person interviews to gain a comprehensive understanding. To improve transferability, the researchers selected districts, hospitals, units and participants using purposive sampling. The researchers also provided detailed descriptions of the participants, environment and methodology. The study procedures, including a thorough explanation of the methodologies and transcripts, were distributed to an impartial individual for analysis and coding to ensure consistent results, thereby increasing confirmability and dependability. Confirmability was further achieved through the involvement of a supervisor and an independent coder with experience to analyse the findings, as well as during the process of the interview, the researcher had to reflect to involve participants in verifying that their viewpoints and experiences are accurately represented.

### Ethical considerations

Ethical clearance to conduct this study was obtained from the University of Venda Research Ethics Committee. The ethical approval number is FHS/23/PDC/01/1409.

The approval was sought from the Limpopo Department of Health Ethics Committee. Additional approvals were sought from the district health offices and the selected hospitals in Limpopo province. The researcher ensured that the ethical norms were followed by maintaining and protecting the participants’ privacy and confidentiality throughout the investigation. The study participants gave their consent freely. Participants and names of hospitals were not identified to ensure anonymity; only codes were used. All soft data were kept secure using a password, whilst hard copies were kept in a locked cupboard.

## Results

### Demographic data of participants

The participants in the study were 24 registered professional nurses, with 20 females and 4 males. The demographic data of the participants are indicated in Table 1. Most participants were female, as nursing is predominantly female.

**TABLE 1:** Demographic data.

No.	Participant	Hospital allocated	Unit allocated	Years of experience	Gender
1.	Participant 1	AA	Paediatrics	30	Female
2.	Participant 2	AA	Paediatrics	28	Female
3.	Participant 3	AA	Maternity	12	Female
4.	Participant 4	BB	Maternity	27	Male
5.	Participant 5	BB	Maternity	20	Male
6.	Participant 6	BB	Paediatrics	12	Female
7.	Participant 7	BB	Casualty	40	Female
8.	Participant 8	BB	Casualty	25	Female
9.	Participant 9	BB	Casualty	30	Female
10.	Participant 10	AA	Paediatrics	25	Female
11.	Participant 11	AA	Maternity	29	Male
12.	Participant 12	AA	Casualty	34	Female
13.	Participant 13	CC	Paediatrics	20	Female
14.	Participant 14	CC	Maternity	10	Female
15.	Participant 15	CC	Maternity	20	Female
16.	Participant 16	CC	Casualty	6	Female
17.	Participant 17	CC	Paediatrics	22	Male
18.	Participant 18	CC	Maternity	10	Female
19.	Participant 19	AA	Maternity	30	Female
20.	Participant 20	BB	Casualty	6	Female
21.	Participant 21	BB	Maternity	9	Female
22.	Participant 22	BB	Paediatrics	10	Female
23.	Participant 23	AA	Casualty	15	Female
24.	Participant 24	AA	Paediatrics	40	Female

No., number.

### Findings

Following a thorough analysis of the transcripts, the researchers identified three main themes and eight sub-themes: administrative, organisational, education and training challenges. The findings from data analysis were in line with the objectives of describing and exploring the experiences of registered professional nurses regarding supervision of nursing. The themes and sub-themes identified by the researcher closely matched those identified by the independent coder. These themes and sub-themes were subsequently discussed, and a consensus was reached on the final themes to be used in the study. The findings were grouped into themes and sub-themes as indicated in Table 2.

**TABLE 2:** Themes and sub-themes.

Themes	Sub-themes
1. Administrative challenges	1.1. Shortage of nursing staff 1.2. Shortage of material resources 1.3. Lack of management support 1.4. Staff attitude and lack of passion for work
2. Education and training challenges	2.1. Lack of staff training 2.2. Lack of supervisory skills and competence
3. Organisational challenges	3.1. Poor working conditions 3.2. Poor infrastructure

### Theme 1: Administrative challenges

Effective nursing care supervision is known to require that no difficulties are encountered in the actual performance of work. Administrative difficulties, however, hindered supervision of nursing care. Administrative issues, including a lack of material resources, inadequate management support, low staff enthusiasm and poor staff attitude, were mentioned by the majority of participants from all selected hospitals. Achempim-Ansong, Kwashie and Ofei (2021) demonstrated that various administrative issues, including poor communication, time constraints, inter-personal conflicts with co-workers, a heavier workload, exhaustion, a lack of motivation from supervisees and a lack of trust, pose challenges for supervision.

Experiences with nursing care supervision in their hospitals were solicited from the registered professional nurses. Next, the participants shared with the researcher the difficulties they faced in supervising nursing care, including a lack of material resources, insufficient staff, a high workload, inadequate staff support and attitude and a lack of enthusiasm.

#### Sub-theme 1.1: Shortage of nursing staff

According to every participant in this study, the department’s biggest problem is a lack of employees. This makes it difficult to supervise nursing care. There was a shortage of nursing staff across all categories, which made it difficult to supervise nursing care and led to higher workloads, exhaustion, burnout and absenteeism. Quotes from participants that highlight the staffing shortfall and its impact on nursing care are included below:

‘In my opinion, the shortage of staff in all nursing categories – from registered professional nurses to registered staff nurses, registered assistant nurses, and general workers – has made my experience with supervision challenging. As a qualified registered professional nurse, I now work alone. I will review the orders from the patients’ files after the doctors have departed, but I will not accompany the doctors on rounds, as I have other obligations.’ (Participant 12, 61 years, female, professional nurses)

‘In the current era, it is extremely challenging to supervise nursing care, and the nursing community is facing a significant staffing shortage due to the department’s inability to hire nurses, primarily because of retirement, death, and nurses leaving for better opportunities.’ (Participant 19, 63 years, female, professional nurse)

#### Sub-theme 1.2: Shortage of material resources

A lack of material resources jeopardises nursing care, as material resources are the tools required to provide nursing care. The second issue affecting nursing care supervision negatively was identified as a lack of material resources. Most of the interviewees stated that their biggest issue whilst supervising nursing care is the lack of material resources. Equipment, stationery, linen and medications were listed as material resources. The following quotations from participants verify this finding:

‘I believe that nursing care supervision is really challenging these days. We lack some of the basic supplies we need to provide high-quality nursing care, such as actual instruments of the trade, including equipment, drugs, and stationery to document the treatment we have provided. As a casualty nurse, you discover that there are no triaging forms. Instead, nurses must draw the forms on blank paper, which makes it difficult to accurately depict all the necessary information.’ (Participant 20, 39 years, female, professional nurse)

‘What I can say is that the level of oversight has decreased since, in the past, material resources were distributed equitably among all wards; however, at the moment, we are facing difficulties related to a lack of supplies, equipment, and medication. When it comes to managing nursing care, all of these material resources are extremely important, yet the unit is operating without them. Delays in maintaining critical equipment, such as ventilators, can occasionally be the reason.’ (Participant 24, 60 years, female, professional nurse)

#### Sub-theme 1.3: Lack of management support

Support from management is crucial to enhancing nursing care supervision. The management must visit every functional area to learn about the difficulties employees face and take the necessary action. The panellists discussed the difficulty of institutional, district and provincial management’s lack of support. The following quotes are from participants regarding the lack of support from management:

‘The management’s lack of assistance causes the staff to be unaware of their problems, which in turn lowers the effectiveness of supervision.’ (Participant 10, 46 years, female, professional nurse)

‘Eish, management does not travel around and assist us these days, which demotivates nurses from performing high-quality work because no one is in charge of the task.’ (Participant 14, 44 years, female, professional nurse)

#### Sub-theme 1.4: Staff attitude and lack of passion for work

Effective nursing care supervision, a quick recovery for patients and positive outcomes were all facilitated by the team’s positive attitudes and enthusiasm. Staff attitudes and a lack of enthusiasm for their work are amongst the difficulties of nursing care oversight, according to the participants. The staff’s attitude and lack of enthusiasm made it difficult to supervise nursing treatment. The following are actual quotes of participants:

‘Currently, the nursing staff – from registered professional nurses to assistant managers – has a negative attitude towards patients, colleagues, and management. They even reach the point where they are unable to communicate effectively with supervisors, patients, and one another. Some nurses exhibit a lack of enthusiasm for patient care, which leads to more patients complaining about the subpar treatment they receive. The efficient supervision of nursing care is also jeopardised by the negative attitude that nurses are displaying.’ (Participant 2, 45 years, female, professional nurse)

‘The attitude of nurses is so poor that they don’t want to work, which makes nursing care oversight difficult. Additionally, they stated that they have a democratic right to refuse to work excessive hours. They also exhibit unfavourable attitudes towards one another, the managers, and the patients. They are constantly displaying resentment.’ (Participant 10, 46 years, female, professional nurse)

### Theme 2: Education and training challenges

The other obstacle to efficient nursing care supervision, according to the participants, is a lack of education and training. The two sub-themes of inadequate education and training are as follows: inadequate supervisory skills and competency and inadequate staff training. One of the key tactics required in the healthcare system, particularly in nursing services, is education and training, as it facilitates the transfer of information and skills. Nursing care monitoring is jeopardised by difficulties with education and training. Lack of supervisory expertise and competency, inadequate staff training and insufficient exposure and empowerment of supervisors are all attributed to difficulties in education and training:

#### Sub-theme 2.1: Lack of staff training

Training of nursing staff is crucial for imparting knowledge and skills to healthcare professionals, particularly in medical and clinical settings. The training referred to here can be either formal or informal, including in-service training. However, many participants cited a lack of staff training as a negative factor in the supervision of nursing care. Participants reported that inadequate training negatively impacted junior nurses, leading to poor performance and attitudes. The following are the direct quotes from participants:

‘Nurses are demonstrating a lack of expertise in their profession, which makes it difficult for them to be supervised. Currently, hospitals offer no in-service training specifically for nursing staff. The problem with supervision is more obvious, though, because nurses lack the training that knowledgeable staff members need to prevent malpractices.’ (Participant 4. 53 years, male, professional nurse)

‘The hospital no longer provides in-service training for nurses, which is problematic since it leads to a lack of knowledge and skills among nurses, which hinders nursing care and monitoring. Because nurses lack information, nursing care supervision is also jeopardised.’ (Participant 14, 44 years, female, professional nurse)

#### Sub-theme 2.2: Lack of supervisory skills and competency

After completing their training, registered professional nurses are required to provide supervision of nursing care. Supervision competency is a critical skill for effective supervision; inadequate supervision skills have a detrimental impact on nursing care supervision. Some registered professional nurses are unable to supervise because they lack supervisory competency. Nurses are trained at various levels within the nursing profession. The following are the direct quotes of participants:

‘Nursing cadres who lack supervisory skills, despite being registered professional nurses, pose a challenge to the nursing profession. Because the supervisors are reported to be unsure of what to supervise, this makes nursing care supervision difficult.’ (Participant 6, 46 years, female, professional nurse)

‘More registered professional nurses who are unfamiliar with supervision pose a challenge to present nursing supervision since they appear to lack a thorough understanding of what they need to monitor. They constantly find themselves moving from one unit to another, suggesting that they lack the necessary supervision abilities.’ (Participant 22, 48 years, female, professional nurse)

### Theme 3: Organisational challenge

For any organisation to perform at its best, it must operate like a system. An organisation cannot perform to its full potential if it faces challenges. Organisational difficulties might lead to problems with nursing care oversight. Sub-themes of organisational problems include inadequate infrastructure and unfavourable working conditions.

Quality nursing care requires a happy practice environment, which also facilitates efficient nursing care oversight. Poor working conditions, inadequate infrastructure and inadequate security systems are examples of organisational issues that jeopardise nursing care supervision. This demotivates employees, which further complicates nursing care supervision. According to some participants, the supervision of nursing care is hampered by organisational issues.

#### Sub-theme 3.1: Poor working conditions

Nursing personnel are motivated by a positive work environment, which also improves staff performance and job satisfaction. Nursing care oversight is enhanced when employees are more satisfied with their jobs and perform better. Participants mentioned that one of the difficulties in supervising nursing care was unfavourable working circumstances. The following are the direct quotes of participants:

‘The bad working conditions in which we as nurses find ourselves jeopardise the supervision that we are expected to offer. The environment in which we operate presents a challenge to nursing care supervision, since you may occasionally find yourself operating under adverse conditions.’ (Participant 8, 42 years, female, professional nurse)

‘The working conditions under which we find ourselves provide a serious challenge to nursing care oversight. Nurses nowadays do not want to work under unsatisfactory conditions. We wish to operate under favourable conditions; thus, these present a challenge to care supervision.’ (Participant 12, 61 years, female, professional nurse)

#### Sub-theme 3.2: Poor infrastructure

A well-maintained infrastructure is essential for both patient care and employee job satisfaction, as both contribute to effective nursing care supervision. The supervision of nursing care is compromised by inadequate infrastructure. The participants noted that it was challenging to oversee nursing care due to insufficient infrastructure. The vessel in which nursing care supervision is conducted is well-built. The following are the personal quotes of the participants:

‘The conditions in which we work are not pleasant due to significant security concerns and decrepit infrastructure, with ceilings falling in some cubicles. When it rains, it pours due to the leaking roofs.’ (Participant 20, 30 years, female, professional nurse)

‘The working conditions are not pleasant because there is a significant difficulty of deteriorating infrastructure, where the ceiling is dropping in some of the cubicles, and it rains due to the leaking roofs.’ (Participant 22, 48 years, female, professional nurse)

## Discussion

The objective of this study was to explore and describe the experiences of registered professional nurses regarding the supervision of nursing care in the selected hospitals of Limpopo Province. This was achieved as the findings of the study indicated the experiences from the perspective of registered professional nurses in their daily responsibility of supervising the nursing care. The study revealed that nursing is compromised by a shortage of nursing staff. The study conducted by Shamsi and Peyravi (2020) concurs with this study regarding Nursing staff shortages as a distinct challenge in Iran. In addition, Mutshatshi and Munyai (2022) also confirmed that globally, a shortage of nursing staff has been a persistent challenge in hospitals, jeopardising nursing care supervision. The severe nursing staff shortage that affects all hospitals was the subject of animated discussions amongst the participants. The severe shortage of staff was cited as a serious challenge in the study conducted by Tamata, Mohammadnezhad and Tamani (2021) in the Republic of Vanuatu. The workforce shortage is further exacerbated by fatigue, which in turn exacerbates burnout and leads to increased absenteeism. Inaccuracy is also a result of employee fatigue, brought on by an increasing workload. Moreover, a greater workload forces registered professional nurses to take on nursing care rather than their supervisory responsibilities, thereby endangering nursing care monitoring. In the study conducted in Iran by Atashi et al. (2023), it was agreed that one of the biggest obstacles to implementing nursing care supervision is a shortage of nurses and supervisors. Rothwell et al. (2021), a study conducted in Egypt, also agreed with participants that a shortage of employees puts pressure on service delivery, leading supervisors to relinquish their responsibilities in a hectic setting.

Waweru, Oluchina and Mwangi (2024) conducted a study in Kenya, indicating that because managers had to shoulder the patient workload, personnel shortages result in a lack of time and impaired nursing care supervision. Nyamhanga, Frumencea and Hurtig (2021), in the study conducted in Tanzania, supported that supervision is hindered by the shortage of healthcare personnel at the level of health facilities, resulting in limitations on the coaching and mentoring of medical experts. Given the above discussion, the various studies conducted concurred with participants that the shortage of nursing staff compromises supervision of nursing care.

The hospital management’s top executive is responsible for ensuring that service delivery is seamless. Support from management is essential because it enables staff members to communicate the challenges they face when delivering nursing care and provides management with direct access to information about staff and carer complaints. Employee satisfaction is ensured when management support motivates nursing personnel to work with joy. A deficiency of managerial support hinders nursing care supervision. A few participants raised the issue of inadequate management support as a reason for the risk to nursing supervision.

Thapa et al. (2022), in a study conducted in Nepal, South Asia, agreed that employees face difficulties due to burnout and a lack of enthusiasm for their work, which leads to supervision challenges when supervisors and senior-level employees fail to provide adequate management support.

Rothwell et al. (2021) also stated that supervisors observed a lack of management support when discussing subpar work performance.

The researchers Saab et al. (2021) concurred that the goal of supervision might be hampered and that clinical practice can be altered by a lack of line management support and experience with clinical supervision. Saab et al. (2021) also support the idea that nursing care oversight is affected by a lack of management support.

It is crucial to expose employees to both formal and informal training, as supervisor training is essential for staff development and capacity building. Insufficient staff training hinders nursing care supervision, as supervisors lose proficiency in effective supervision methods. Some participants believe that inadequate staff training hinders the supervision of nursing care, as seen by staff members becoming disinterested in their work due to constant complaints about their lack of training. Some participants claimed that since they had been waiting a long time for training and nothing had come of it, the highly specialised stations were now being managed by unspecialised registered professional nurses.

Also, several participants said that even in-service training doesn’t help them become more capable. Rothwell et al. (2021) stated that insufficient supervisor training hindered oversight and led to inefficiency.

Abujaber et al. (2022) claimed that supervisors’ lack of formal training in supervision hindered their capacity to effectively supervise nursing care and to direct the growth and performance of employees, thereby negatively affecting nursing care supervision. Terry et al. (2020) also note that supervisors who have received little to no training in effective supervision face difficulties in assuming the role, which, in turn, leads to challenges in effectively supervising nursing care.

Supervisory ability and skill are critical for managing and executing nursing care supervision. All certified professional nurses must meet these requirements to supervise nursing care effectively. Insufficient supervisory competencies and skills hinder nursing care oversight. The incapacity of some registered professional nurses to manage situations indicated that they lacked supervisory skills and expertise, according to the participants.

Rothwell et al. (2021) suggested that a supervisor’s incapacity to deal with various personality types, become intolerant, blameful and inflexible, or refuse to accept the task at hand, such as supervising, are all indications of a lack of competence and skills.

Rothwell et al. (2021) furthermore stated that supervisors are said to lack supervisory abilities and competence if they are unfamiliar with professional guidelines (i.e. standards imposed by regulators), their role and obligations as supervisors, ethical standards set by employers and inadequate educational preparation. All of this contributes to insufficient nursing care oversight.

To motivate employees to effectively carry out their supervision duties, the environment must be friendly. Unfavourable working conditions can endanger the lives of employees and patients, making it more difficult to provide effective nursing care.

Unfavourable workplace circumstances, according to participants, make it challenging to supervise nursing care, which demotivates staff.

Gheshlagh et al. (2024), in the study conducted in Iran, agreed with participants that a lack of supportive and friendly work environments may hinder nurses’ ability to manage nursing care, affecting their health and communication skills. Tenza et al. (2024) demonstrated that poor work practices and a stressful work environment contribute to mental health issues and increased employee turnover.

The facility must provide nursing care in good condition. Inadequate infrastructure hinders nursing care supervision and patient care. According to some participants, nursing care supervision is challenging when there is insufficient infrastructure. A shortage of wards to admit patients, as well as leaky roofs, broken windows and ceilings, were amongst the problems they mentioned related to the insufficient infrastructure. Atashi et al. (2023) said that inadequate infrastructure, underqualified staff and poorly written job descriptions and rules make it impossible to expect high-quality monitoring.

In conclusion, the global and local literature reviewed concurred with the findings of this study, namely, experiences of registered professional nurses regarding supervision of nursing care and the challenges encountered in supervision of nursing care, and there was no literature with findings contrary to the study’s findings.

### Limitations

The study was conducted in the selected hospitals of Limpopo Province; therefore, the findings of this study cannot be generalised to other hospitals, districts and provinces. The scope is around Limpopo Province. During the data collection process, some managers were not interested in participating in the study; only registered professional nurses and operational managers participated. The other limitation the researcher encountered was the unavailability of a sufficient number of participants due to staff shortages.

### Recommendations

The study highlighted the experiences of registered professional nurses regarding supervision of care in the selected hospitals of Limpopo Province. The effective implementation of supervision of nursing care depends on multiple factors, such as addressing administrative, organisational, educational and training challenges. The addressing of these findings has the potential to change the nursing practice. Ongoing in-service training and workshops should be implemented regularly to keep registered professional nurses updated on supervision of care. Training of trainers would ensure the sustainability of training and the continuous supervision of nursing care. Additionally, addressing resource shortages is crucial; management should prioritise resource allocation, furthermore, conducting future studies on how to effectively implement supervision of nursing care in other provinces and hospitals.

## Conclusions

The study aimed to investigate and describe the experiences of registered professional nurses in supervising nursing care. The findings have a negative impact on the supervision of nursing care by registered professional nurses; consequently, there is a need to address these findings to improve the supervision of nursing care, thereby improving patient outcomes and minimising complaints.
